# Bibliometric Analysis on Enamel Remineralization Agents

**DOI:** 10.7759/cureus.105086

**Published:** 2026-03-12

**Authors:** Sangaragomathy A M, Shruthi Eshwar, Aarya N Bharadwaj, Nikhil V Suresh, Srivastava B K, Lakshmi J, Ishan Mukherji

**Affiliations:** 1 Public Health Dentistry, KLE Society's Institute of Dental Sciences, Bengaluru, IND; 2 Public Health Dentistry, Guru Nanak Institute of Dental Sciences and Research, Kolkata, IND

**Keywords:** bibliometric analysis, bioactive glass, biomimetic agents, cpp-acp, enamel remineralization, fluoride, nanohydroxyapatite, preventive dentistry

## Abstract

Dental caries remains a major global oral health concern, affecting almost 2.5 billion people. While restorative treatments remain widely used, modern dentistry increasingly focuses on preventive and minimally invasive approaches that strengthen the natural process of enamel remineralization. A comprehensive literature search was conducted in PubMed for enamel remineralization agents from 2015 to 2025. Clinical trials, randomized controlled trials, systematic reviews, and meta-analyses published in English were included.

After screening, 153 publications were retained. RStudio (Biblioshiny package for the Bibliometrix tool, Posit Software, Boston, MA) was used to evaluate publication trends, citation impact, keyword structure, and collaboration networks. A total of 153 publications from 68 sources were identified, contributed by 700 authors, with an average annual growth rate of 10.65% and a mean document age of 4.58 years. Two waves of research activity were observed, with fluoride and casein phosphopeptide-amorphous calcium phosphate (CPP-ACP) dominating earlier years and biomimetic agents emerging more recently. This bibliometric mapping reveals enamel remineralization research as a field characterized by steady growth, global collaboration, and diversification of agent types. Fluoride and CPP-ACP continue to anchor the evidence base, while nanohydroxyapatite, bioactive glass, peptides, and natural products are emerging as promising contenders, driving the second wave of publications.

## Introduction and background

Dental caries persists as one of the world’s most common chronic diseases, affecting almost 2.5 billion people, thereby creating a significant public health burden [1]. It arises due to an imbalance between enamel demineralization and remineralization, largely mediated by acidogenic bacterial biofilms [2]. While restorative treatments remain widely used, modern dentistry increasingly focuses on preventive and minimally invasive approaches that strengthen the natural process of enamel remineralization [3].

Preventive remineralization strategies are particularly relevant in pediatric dentistry, where early intervention is critical for controlling the progression of early childhood caries and preserving tooth structure. Fluoride sets the benchmark of enamel remineralization by forming fluorapatite - a more durable, acid-resistant variant - thereby reducing demineralization rates [4]. However, lately the scope of research has expanded to include a variety of innovative biomimetic alternatives.

Casein phosphopeptide-amorphous calcium phosphate (CPP-ACP) stabilizes calcium and phosphate ions at the tooth surface to boost remineralization [5]. Bioactive glass (especially nano-sized formulations) has shown strong enamel-hardening potential, with recent studies demonstrating superior recovery of enamel microhardness compared with fluoride varnish and CPP-ACP [6]. Peptide- and protein-based agents imitate enamel matrix proteins and salivary peptides, supporting crystal nucleation and growth [7]. Natural biopolymers such as chitosan, green tea catechins, and propolis have gained interest due to accessibility and biocompatibility [8]. Nanohydroxyapatite (nHAP) is an emerging biomimetic agent for enamel remineralization due to its structural similarity to enamel apatite [9,10].

The expanding scope of these approaches in recent times calls for a comprehensive overview to compare the research trajectories across agents. While systematic and narrative reviews focus on the clinical efficacy of individual agents, broader mapping of scientific output remains limited. Bibliometric analysis is a quantitative method used to analyze large volumes of scientific literature through publication and citation data, enabling researchers to identify publication trends, influential contributors, and the intellectual structure of a research field [11]. Bibliometric analysis provides an objective approach to quantify publication trends, citation impact, and thematic evolution within a research field [11].

Therefore, this study applies bibliometric methods to analyze research on enamel remineralization indexed in PubMed. It aims to identify publication patterns, leading contributors, and thematic clusters to provide a bird’s-eye view across categories of remineralization agents, establishing a structured overview that can inform future research and clinical innovation.

## Review

Data source and search strategy

PubMed was used to conduct a comprehensive search on enamel remineralization agents. Keywords such as “enamel remineralization”, “fluoride”, “CPP-ACP”, “bioactive glass”, “nanohydroxyapatite”, and related terms were used, in combination with Boolean operators such as AND, OR, to refine the results.

Filters applied included: 1. Timeframe: 2015-2025. 2. Article type: clinical trials, randomized controlled trials, meta-analyses, and systematic reviews (narrative reviews, books, and documents were excluded). 3. Language: English.

Data extraction and cleaning

The initial PubMed search yielded 182 records. Titles and abstracts were screened to exclude studies not relevant to enamel remineralization, such as those focusing solely on dentin or unrelated preventive interventions. Additional exclusions were applied for duplicate entries and studies that did not meet the predefined eligibility criteria (e.g., non-English-language publications, narrative reviews, books, or documents). After this screening process, 153 publications were retained for the final analysis.

Analytical tools

All citation records were retrieved from PubMed in .nbib format and subsequently converted into BibTeX format for compatibility. The bibliometric analysis was performed using RStudio (Biblioshiny package for the Bibliometrix tool, Posit Software, Boston, MA). This enabled descriptive analysis (annual scientific production, most productive authors, journals, and countries), citation impact assessment, trend analysis, and keyword mapping. Thematic evolution and network visualization were also carried out using Biblioshiny’s integrated functions.

General bibliometric overview

**Figure 1:**
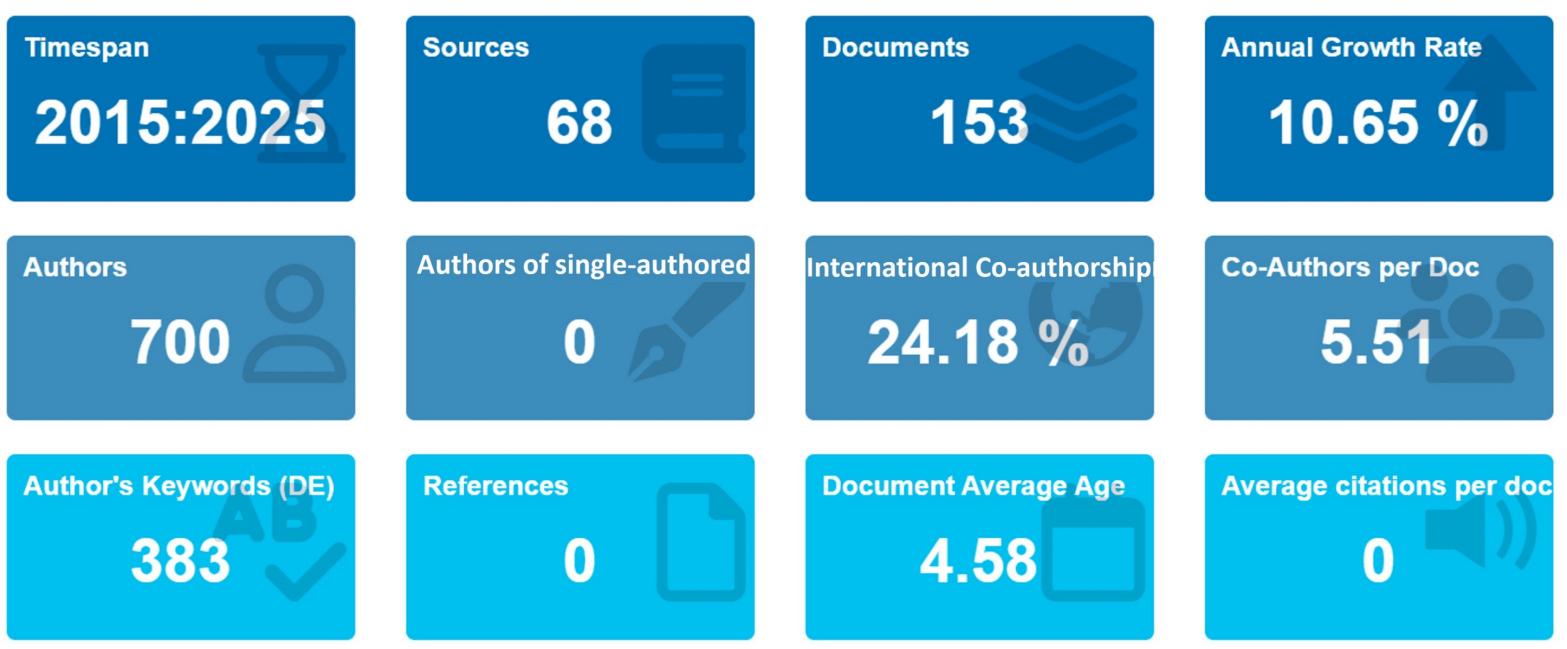
Overview of Key Bibliometric Indicators

Between 2015 and 2025, a total of 153 publications were retrieved from 68 sources, contributed by 700 authors. The average annual growth rate of publications in this field was 10.65%. The average document age was 4.58 years, suggesting that most contributions are relatively recent. Notably, no single-authored papers were identified, and each document had an average of 5.51 co-authors, reflecting the strong collaborative character of this field. International partnerships accounted for 24.18% of publications, highlighting the global relevance of this domain. Furthermore, 383 unique keywords were indexed, suggesting broad thematic diversity across different remineralization agents and approaches (Figure 1).

Descriptive trends

**Figure 2:**
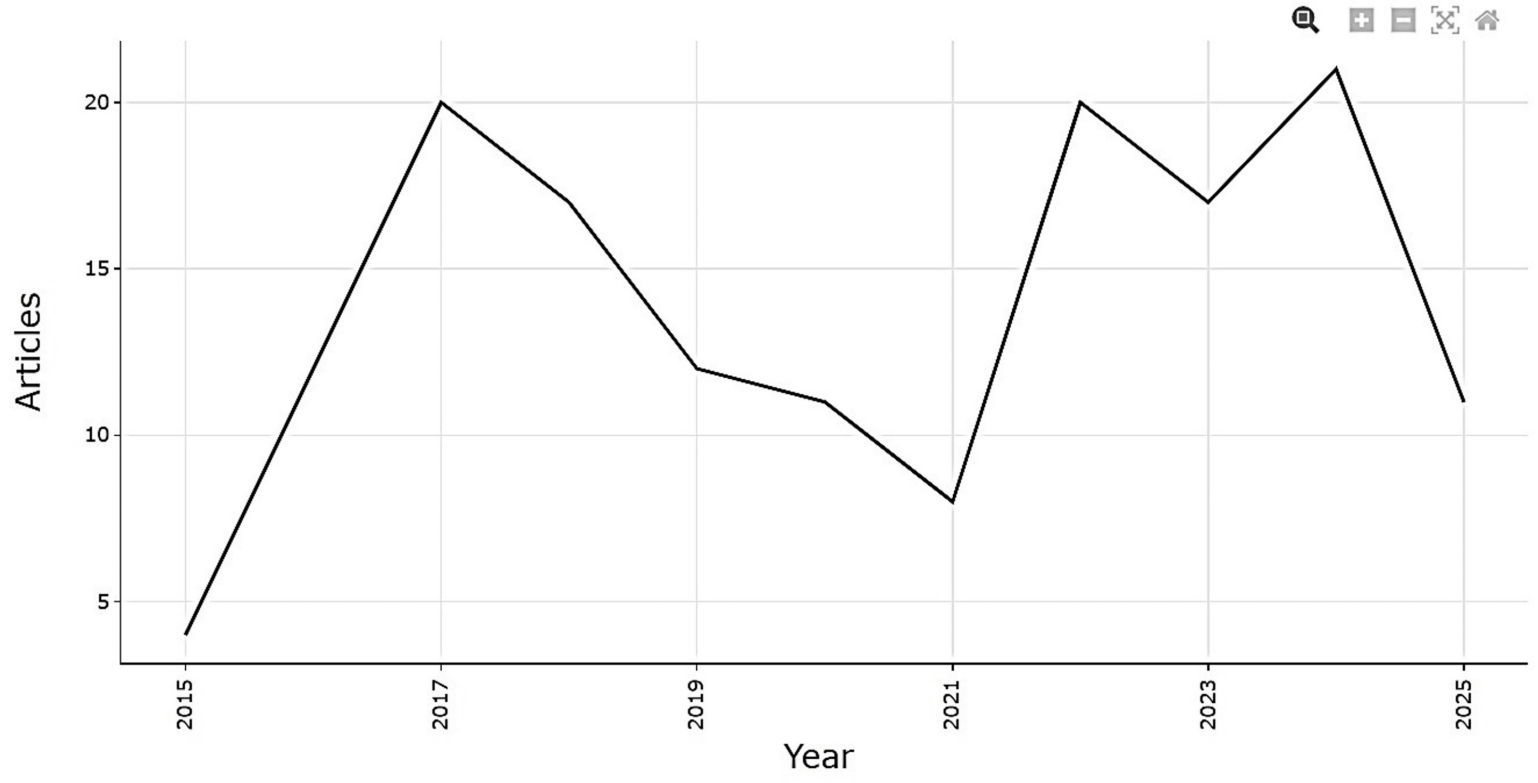
Annual Scientific Production (2015-2025)

The dataset comprised 153 publications over the timespan 2015-2025, with an average annual growth rate of 10.65%. As illustrated in Figure 2, annual scientific production showed two distinct waves of activity. The first surge occurred between 2015 and 2017, during which output increased from 4 articles in 2015 to a peak of 20 in 2017, reflecting rapid initial interest in enamel remineralization research, particularly around established agents such as fluoride and CPP-ACP. This was followed by a gradual decline from 2018 to 2021, reaching a low of eight publications, possibly due to topic saturation or shifting research priorities. From 2022 onwards, a second wave emerged, culminating in a peak of 21 publications in 2024, likely driven by diversification into newer approaches such as nHAP, bioactive glass, peptides, and natural products. The apparent decline in 2025 (11 articles) is most likely attributable to incomplete indexing for the current year rather than a true reduction in research activity. Overall, the trajectory highlights enamel remineralization as a dynamic and evolving field, where the first wave was dominated by core agents, while the second wave reflects the rise of innovative and emerging categories.

Most productive contributors

Sources (Journals)

**Figure 3:**
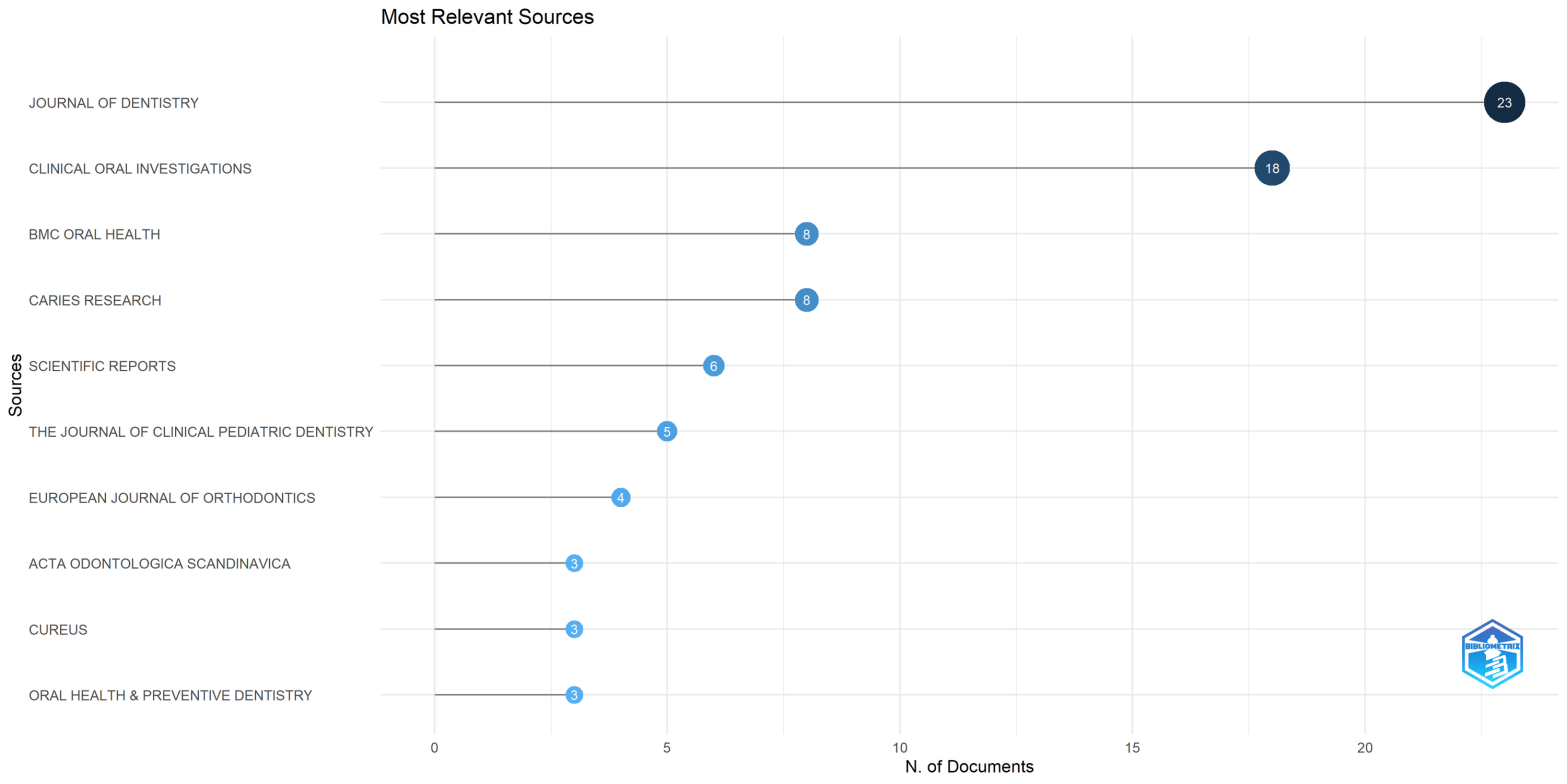
Most Relevant Sources (Journals)

Analysis of source distribution revealed that research on enamel remineralization was concentrated in a small number of leading journals. The most productive outlets were the Journal of Dentistry (23 articles) and Clinical Oral Investigations (18 articles), followed by BMC Oral Health (8 articles), Caries Research (8 articles), and Scientific Reports (6 articles) (Figure 3).

**Figure 4:**
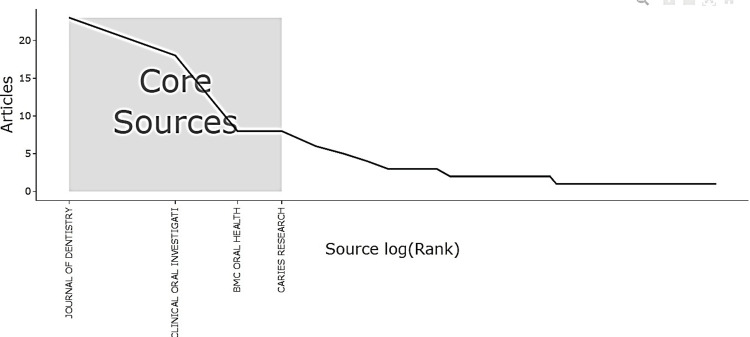
Core Sources by Bradford’s Law

The Bradford’s law plot (Figure 4) confirms that these journals form the core sources - led by Journal of Dentistry and Clinical Oral Investigations - that account for the majority of publications in enamel remineralization research.

Authors

**Figure 5:**
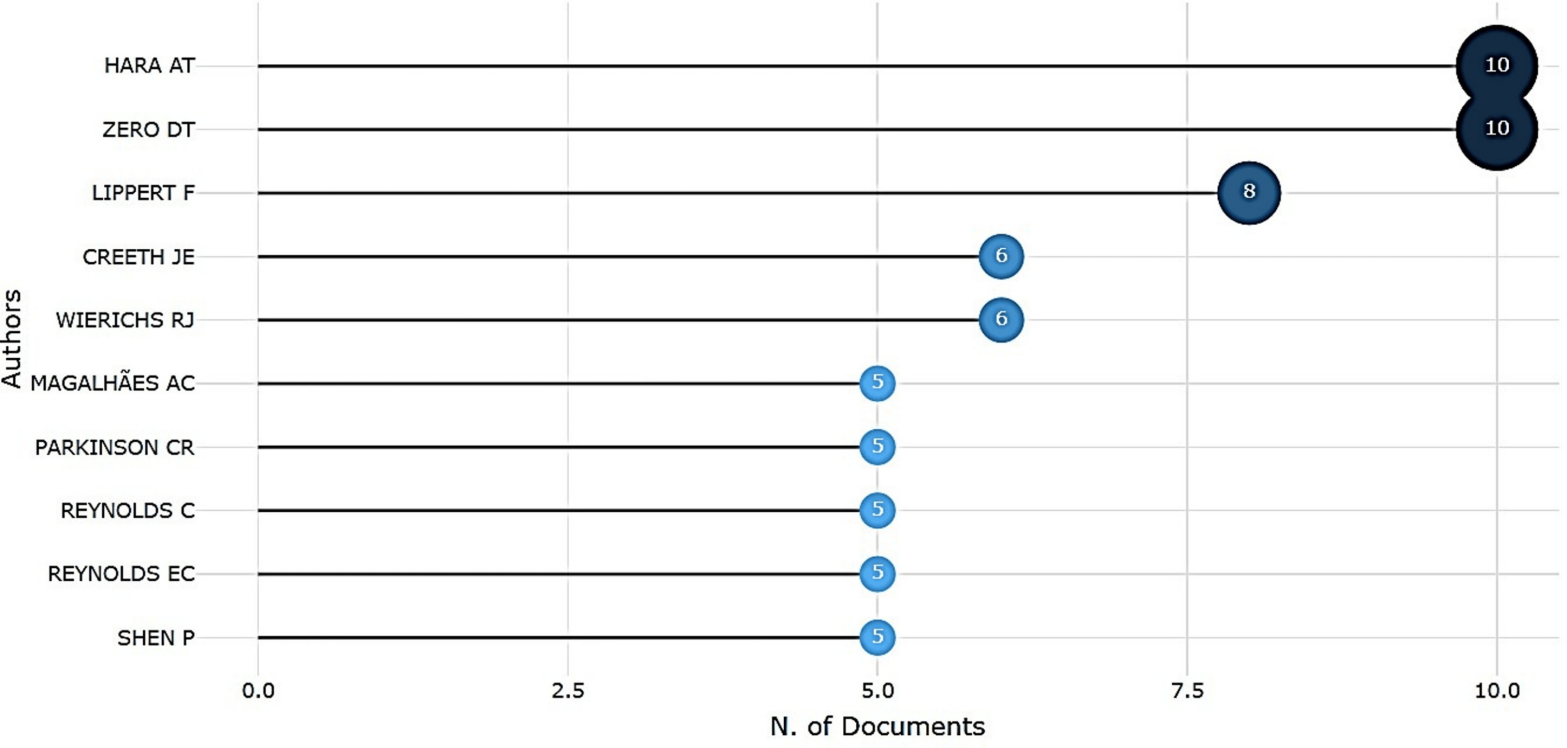
Most Relevant Authors

**Figure 6:**
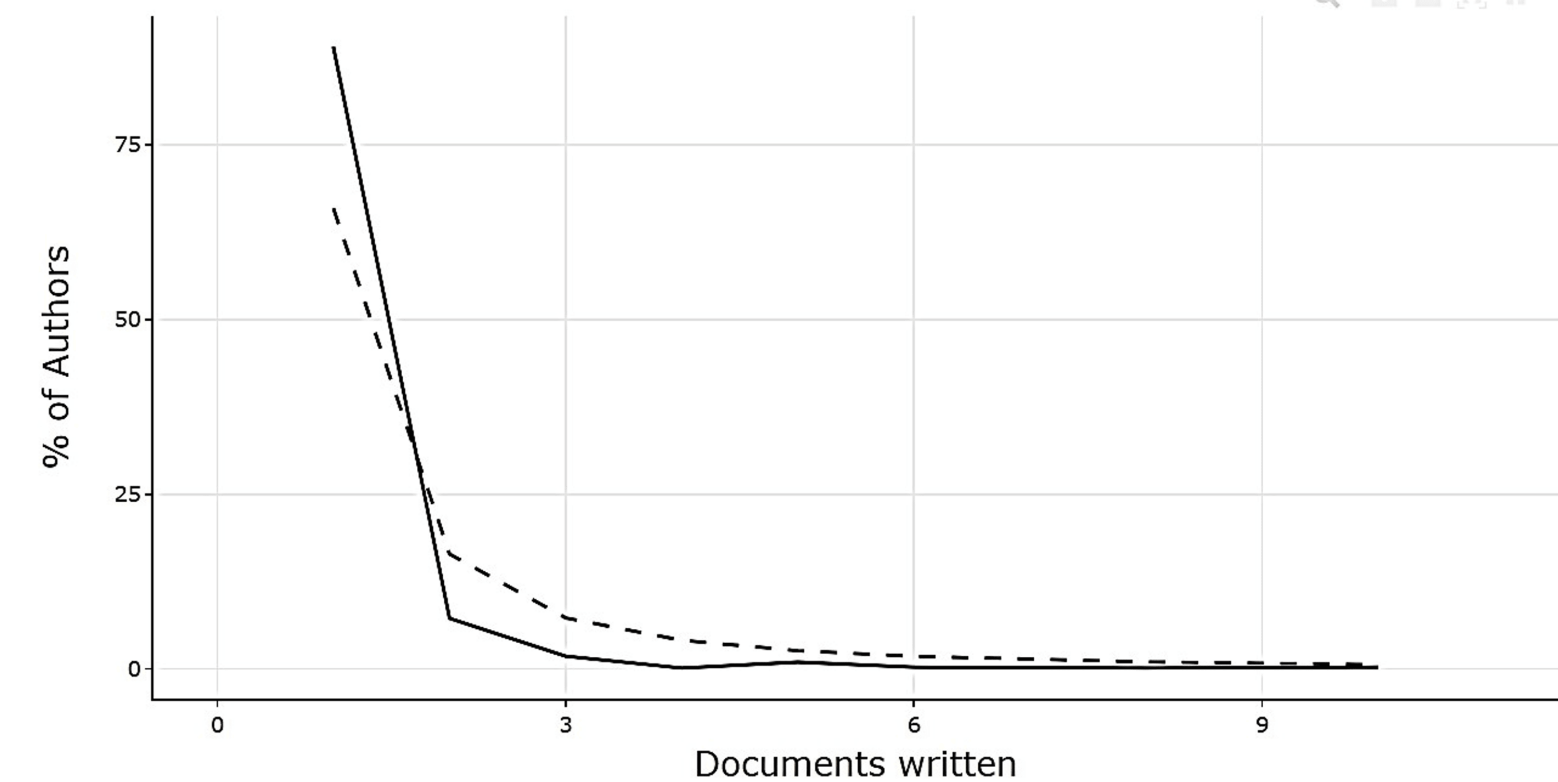
Author Productivity Through Lotka’s Law

Among authors, Hara AT and Zero DT led with 10 publications each, followed by Lippert F, Creeth JE, and Wierichs RJ (Figure 5). Author productivity analysis using Lotka’s Law indicated that most authors contributed only one paper, while a small group produced multiple works, consistent with theoretical expectations (Figure 6).

Institutions (Affiliations)

**Figure 7:**
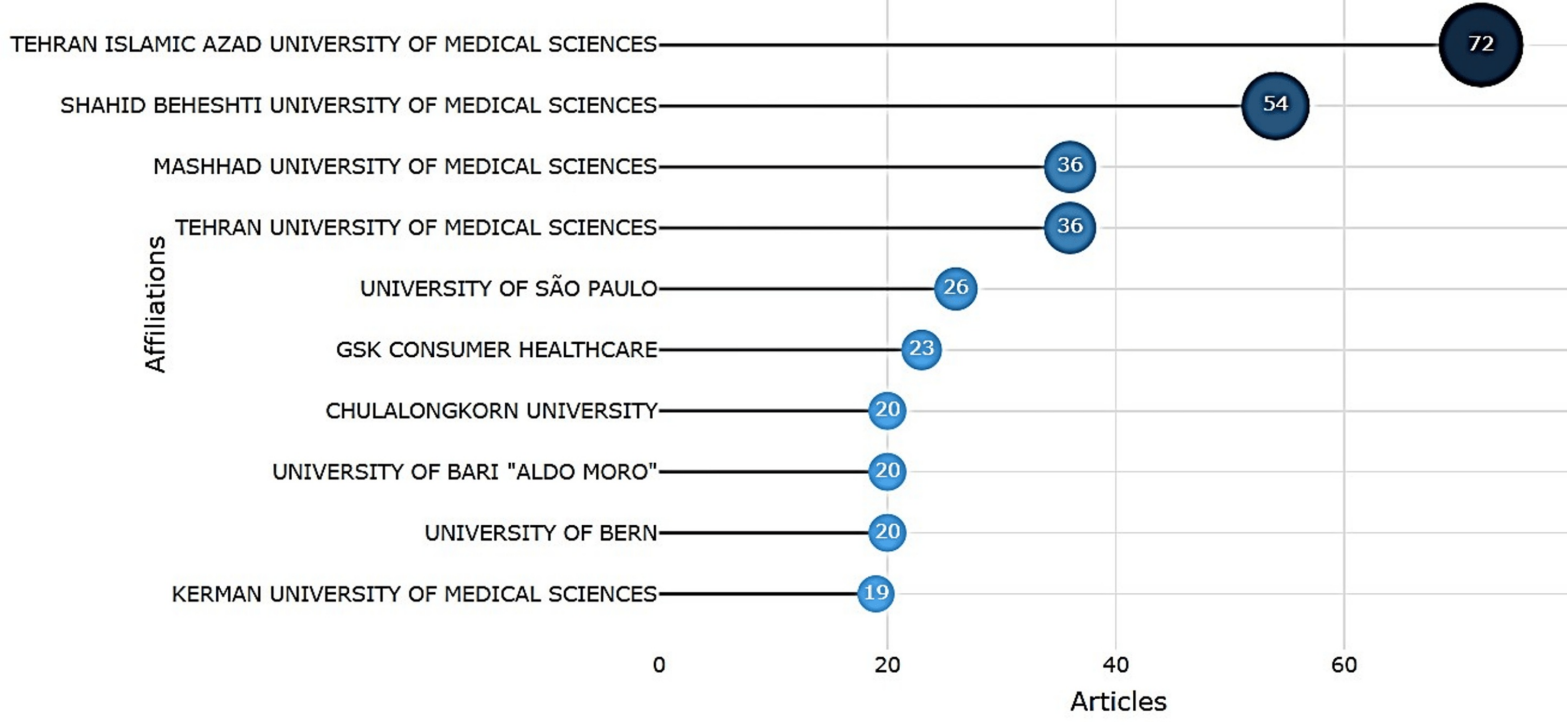
Most Relevant Affiliations

Institutional contributions were led by affiliations from Iran, Brazil, and Europe. The top 10 institutions are illustrated in Figure 7.

Countries

**Figure 8:**
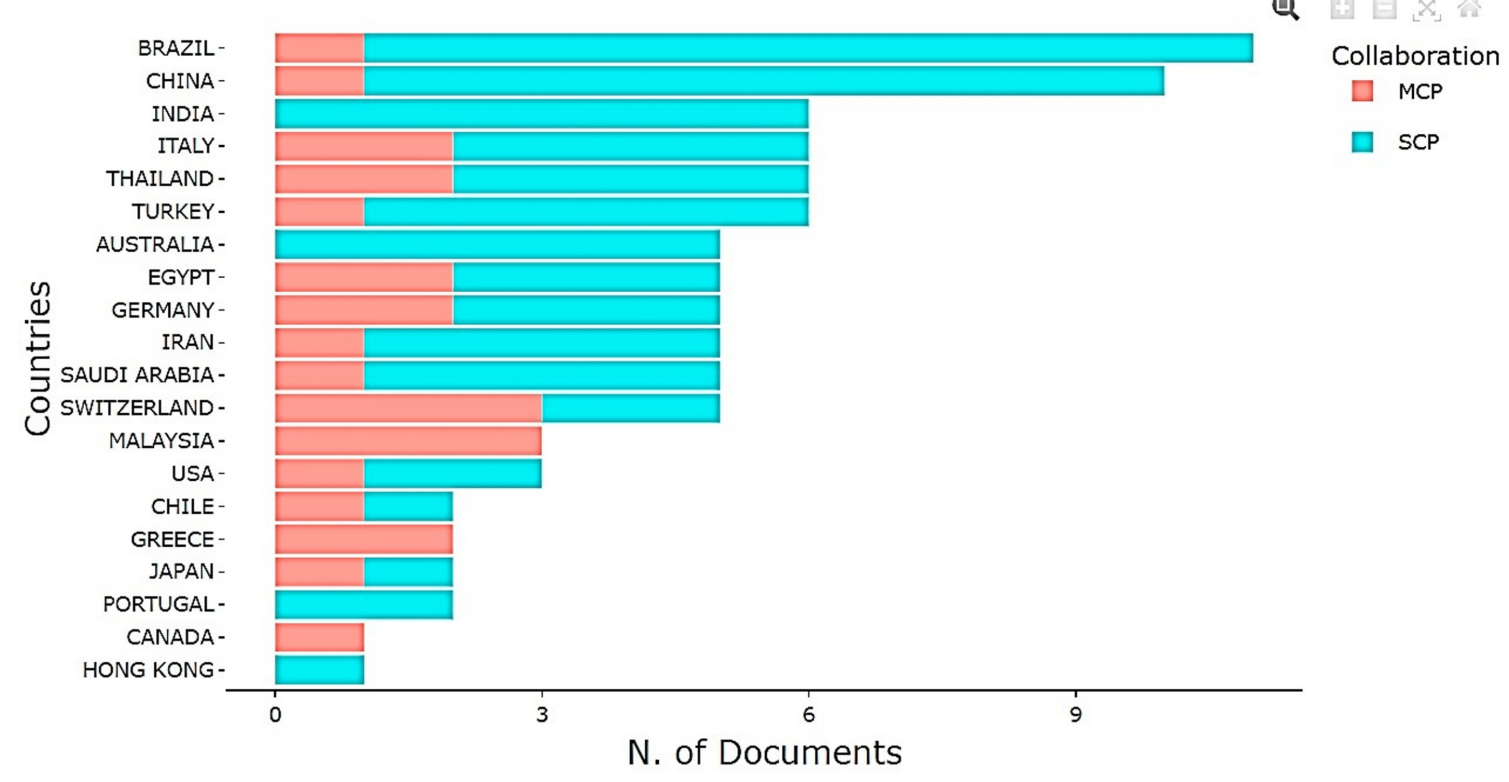
Most Relevant Countries

Geographically, Iran dominated publication output, followed by Brazil, China, Italy, and India. This distribution reflects both emerging and established research hubs (Figure 8).

Keyword Analysis

**Figure 9:**
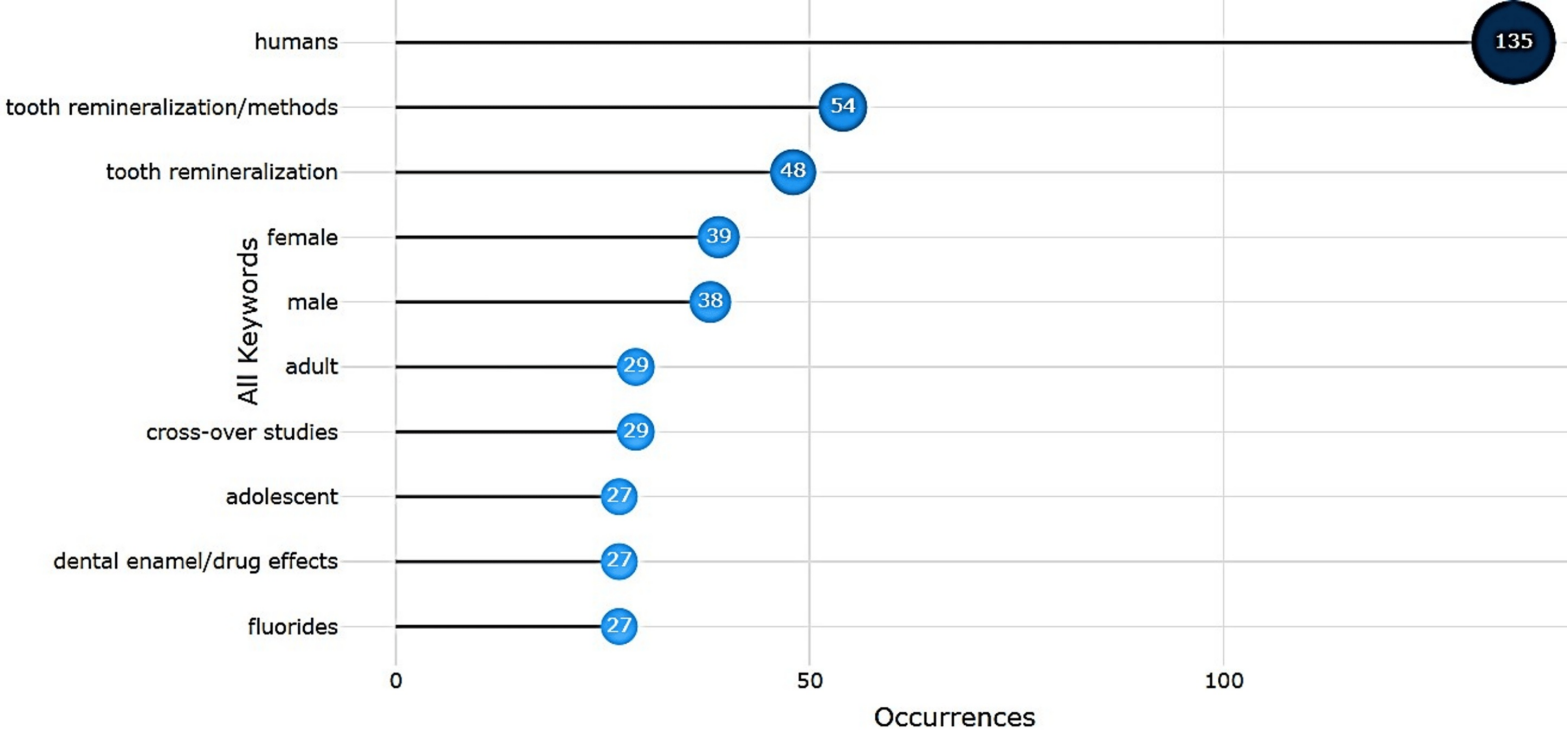
Most Frequent Terms in Keyword Analysis

**Figure 10:**
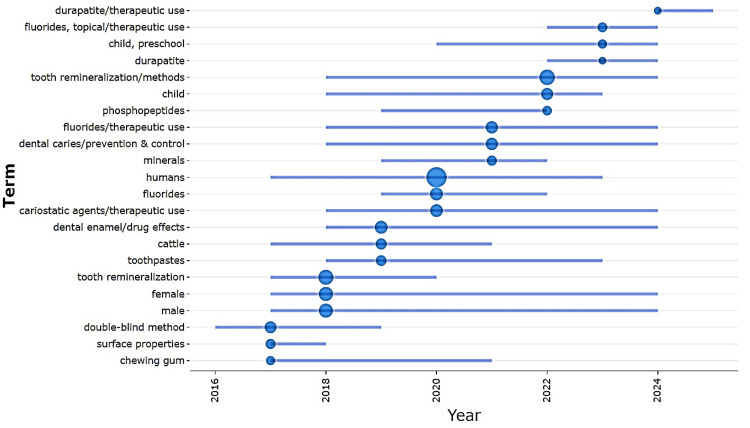
Trend Topic Analysis

Keyword analysis highlighted the clinical orientation of the literature. The most frequent terms were “humans” (135 occurrences), “tooth remineralization/methods” (54), and “tooth remineralization” (48) (Figure 9). Other recurring descriptors included population identifiers such as “female” (39) and “male” (38), reflecting the clinical and trial-based focus of many studies. Trend topic analysis (Figure 10) revealed a shift from methodological studies (2016-2018) to applied clinical strategies (2017-2021), with more recent emphasis on biomimetic and natural agents.

Conceptual Structure Analysis: Network Approach and Factorial Approach

**Figure 11:**
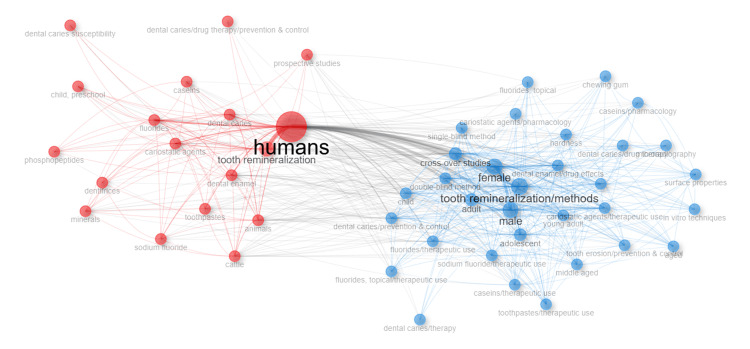
Co-occurrence Network

The keyword co-occurrence network (Figure 11) demonstrated two main research clusters. One cluster was dominated by clinical descriptors (“humans,” “male,” “female”) and study designs, while the other grouped around technical and agent-related terms such as “tooth remineralization/methods,” “fluorides,” and “crossover studies.” This division highlights how the field simultaneously addresses clinical application and agent-based innovation.

**Figure 12:**
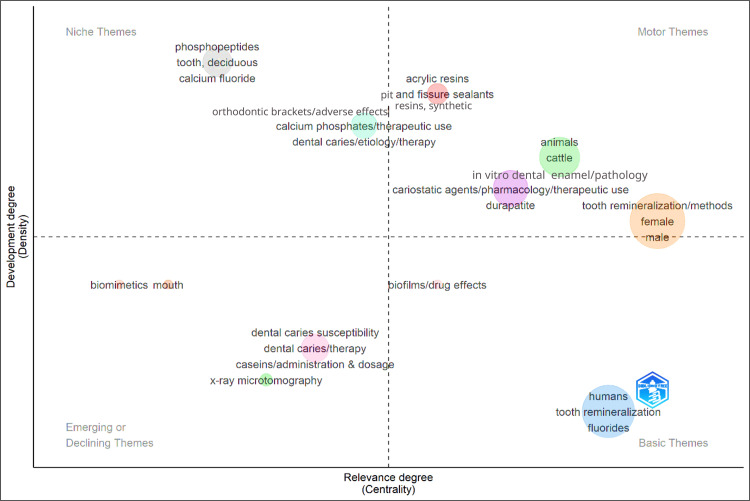
Thematic Analysis

The thematic map (Figure 12) positioned fluoride and tooth remineralization as basic themes, reflecting their central and foundational role. Motor themes included “tooth remineralization/methods,” “sealants,” and trial-related terms, indicating that comparative evaluation of agents (fluoride vs. CPP-ACP, bioactive glass, nHAP) remains a highly developed and relevant area. Niche themes such as “phosphopeptides” and “calcium fluoride” suggest specialized but targeted exploration of peptide-based or modified fluoride systems, while emerging/declining themes such as “biomimetics” point to growing interest in novel biomimetic agents and natural products.

**Figure 13:**
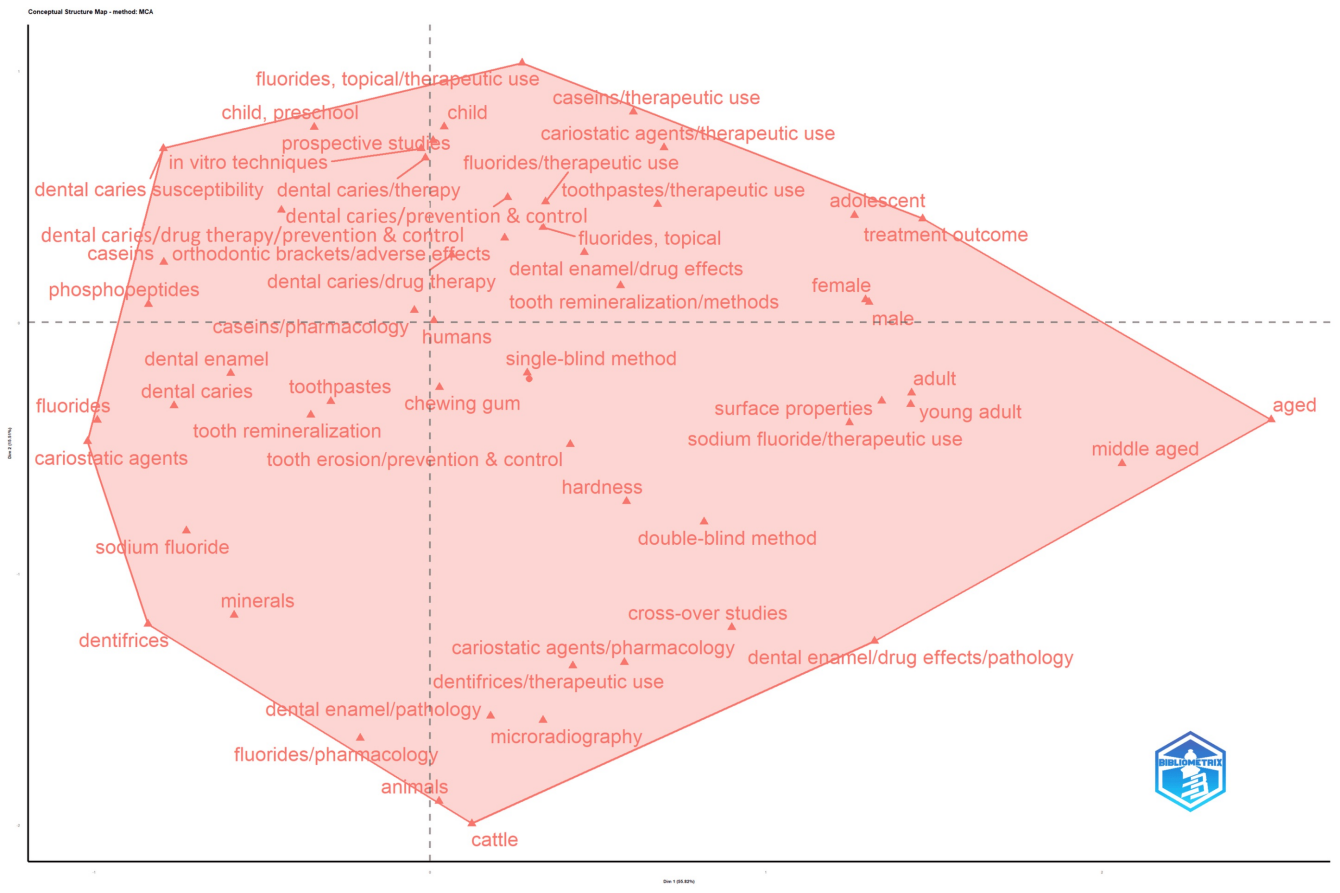
Multiple Correspondence Analysis

The multiple correspondence analysis conceptual structure map (Figure 13) illustrates the organization of the literature based on the co-occurrence of indexed terms. Preventive and remineralization concepts occupy the left and central regions, with adjacent casein- and phosphopeptide-related descriptors indicating a related protein-based remineralization theme. Fluoride-associated terms are distributed across the map, reflecting integrative links with preventive, clinical, methodological, and population dimensions rather than a single cluster. Laboratory assessment methods, enamel material properties, and clinical trial designs extend toward the lower central and right regions, while demographic and treatment outcome descriptors extend toward the far right region. Overall, the map depicts the relational structure connecting preventive strategies, biological mechanisms, methodological evaluation, and population characteristics within remineralization research.

Social Structure Analysis: Collaboration Pattern

**Figure 14:**
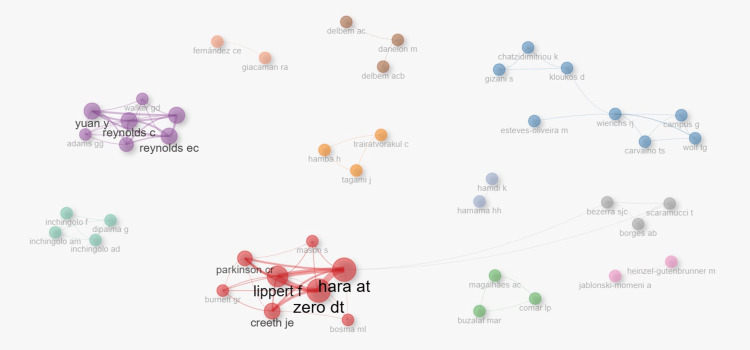
Collaboration Network

The author collaboration network (Figure 14) revealed distinct clusters of researchers contributing to enamel remineralization research. Prominent groups included Hara AT, Lipert F, Zero DT, Creeth JE, and Parkinson CR, who formed a central cluster with strong co-authorship links. Yuan Y, Reynolds C, and Reynolds EC represent another highly productive group centered around CPP-ACP studies. Smaller but cohesive clusters were identified for Delbem AC, Buzalaf MAR, and Bezerra S, indicating emerging or regionally concentrated collaborations. This clustering highlights the dominance of a few influential author groups while pointing to opportunities for broader cross-cluster collaboration.

**Figure 15:**
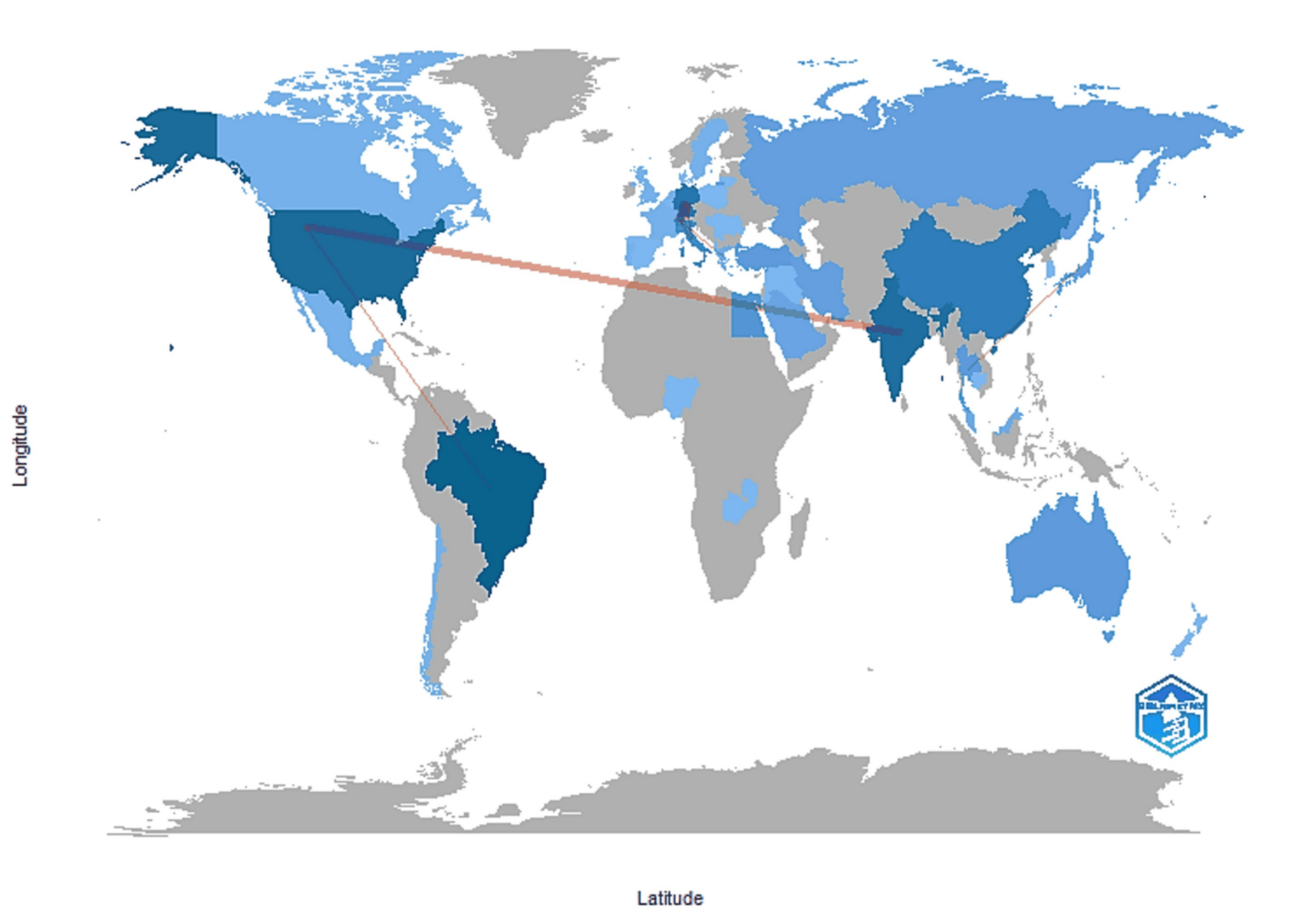
Countries' Collaboration World Map

The country collaboration map (Figure 15) demonstrated that enamel remineralization research is highly internationalized. Strong partnerships were observed between the United States, Brazil, India, and several European countries, while Asian countries such as China and Japan also showed significant contributions. Approximately one-quarter of publications involved international co-authorship, reflecting the global and collaborative nature of the field.

Discussion

This bibliometric analysis mapped global research trends on enamel remineralization agents over the past decade (2015-2025). The results demonstrate a dynamic and evolving field, with two clear waves of research activity. The first wave, dominated by fluoride and CPP-ACP, reflects their historical role as foundational agents in preventive dentistry. Fluoride remains the most extensively studied agent, with high citation impact due to its long-standing use and evidence base. CPP-ACP, emerging as a biomimetic adjunct in the early 2000s, consolidated its relevance during the first growth phase. The second wave, visible from 2022 onwards, was driven by diversification into nHAP, bioactive glass, peptide/protein-based approaches, and natural products. While these categories currently contribute fewer publications than fluoride, their upward trajectory in output and presence as “motor themes” in conceptual maps indicate growing scientific interest. nHAP and bioactive glass, in particular, are gaining visibility due to their biocompatibility, bioactivity, and potential as alternatives or complements to fluoride. Natural products (e.g., chitosan, plant-derived polyphenols) and peptide-based strategies represent emerging areas with promise for translation into minimally invasive, patient-friendly solutions. The social and conceptual structure analyses highlight the collaborative and global nature of this research, with strong author clusters (e.g., Hara, Reynolds, and Delbem) and international partnerships bridging the United States, Brazil, India, and Europe. However, the dominance of a few clusters suggests potential research silos, emphasizing the need for broader interdisciplinary and cross-country collaborations to accelerate comparative trials across agents. Overall, when interpreted in light of existing research, the present findings confirm an evolving field that is transitioning from a fluoride-centered foundation to a more diverse, biomimetic, and innovation-driven research landscape, while still requiring robust long-term clinical validation for emerging agents.

Future directions

Future directions in enamel remineralization research should focus on strengthening clinical relevance, individualization, and public health impact. Much of the existing evidence is derived from short-term or in vitro studies, which limit conclusions about durability and real-world effectiveness. Long-term, in vivo investigations are therefore necessary to establish sustained clinical benefits under routine oral conditions. In parallel, research should increasingly adopt a personalized preventive approach that accounts for patient-specific factors such as saliva composition, oral microbiome characteristics, and genetic susceptibility, allowing better-tailored remineralization strategies to individual risk profiles. Finally, future work should support policy and public health integration by using comparative and bibliometric evidence to guide the development of cost-effective preventive guidelines, particularly in low- and middle-income country settings where affordable non-fluoride alternatives may improve access to preventive oral care.

Limitations include a PubMed-only search, screening English-only records, and citation lag for recent publications.

## Conclusions

This bibliometric and comparative mapping reveals enamel remineralization research as a field characterized by steady growth, global collaboration, and diversification of agent types. Fluoride and CPP-ACP continue to anchor the evidence base, while nHAP, bioactive glass, peptides, and natural products are emerging as promising contenders, driving the second wave of publications. Conceptual and social network analyses confirm both the foundational role of traditional agents and the growing visibility of innovative biomimetic strategies. Future research must prioritize comparative, clinical, and interdisciplinary investigations to consolidate evidence for next-generation remineralization agents and guide their integration into preventive dentistry practice.
